# Communication disorders in subjects with normal hearing: a behavioral and electrophysiological study

**DOI:** 10.5935/1808-8694.20130012

**Published:** 2015-10-14

**Authors:** Marta Regueira Dias Prestes, Maria Angela Guimarães Feitosa, André Luiz Lopes Sampaio, Maria de Fátima Coelho Carvalho

**Affiliations:** PhD student at the University of Brasília (Speech and Hearing Therapist in the Auditory Health Sector of the Brasília University Hospital); PhD in Psychology (Psychobiology) by the University of Michigan (Professor at the Institute of Psychology of the University of Brasília); PhD by the University of Brasília (ENT in the Cochlear Implant Program of the Brasília University Hospital); Specialist in Audiology by the University of Franca (Speech and Hearing Therapist in the Auditory Health Sector of the Brasília University Hospital). University of Brasília - UnB

**Keywords:** audiometry, audiometry, evoked response, communication disorders, self-assessment

## Abstract

Hearing thresholds are not always predictive of performance in environments with reduced extrinsic redundancy.

**Objective:**

To investigate the communication disorders reported by adults with normal hearing, and to assess their underlying conditions through behavioral and electrophysiological testing.

**Method:**

This case control study enrolled 20 adults with normal hearing thresholds and divided them into two groups: a case group with 10 adults with hearing impairment-related communication disorders and a control group with 10 adults with normal hearing. The frequency of occurrence of communication difficulties was recorded during speech recognition tests run in quiet and noisy conditions, audiometry, and auditory evoked brainstem potential testing.

**Results:**

Case group subjects differed statistically from controls only in self-reported scores of hearing impairment. The groups did not differ in the other ratings. A positive correlation was found between hearing thresholds and scores on self-reported impairment.

**Conclusion:**

The combination of hearing complaints and unaltered audiograms was not correlated with differences in performance in speech recognition testing in noisy conditions or in the remaining evaluations. Correlation analysis showed that the higher the pure tone thresholds, the greater were the reported communication difficulties, even in thresholds between 0 and 25 dB.

## INTRODUCTION

Self-reporting is an important tool in the investigation of communication difficulties subjects experience in their everyday lives^1^. A wide range of self-assessment scales are available to evaluate hearing impairment, but they were developed to meet the various needs related to the auditory rehabilitation process, and include matters such as one's adaptation to using hearing aids. Given the absence of validated instruments to analyze people with normal hearing, those interested in looking into the hearing difficulties of the population in general resort to these scales, though they were developed to meet the needs of a specific population^1^^,^^2^.

Studies indicate that hearing thresholds can be used to predict speech recognition performance in quiet conditions^3^^,^^4^, but not in environments with reduced extrinsic redundancy such as noisy areas^5^. This is not a recent finding. In 1970, Carhart & Tillman suggested that communication handicaps could be quantified not only by measurements of pure-tone sensitivity and speech recognition in quiet conditions, but also by speech recognition testing in the presence of noise^6^.

A study compared the performance of subjects submitted to speech reception tests in quiet and noisy conditions, and found that individuals with hearing loss could be divided into two groups: one with subjects with hearing loss characterized by attenuation, i.e., reduced levels of sound stimuli (speech and noise), and one with individuals with hearing loss characterized by distortion, i.e., reduced signal to noise ratios^5^. It was also found that a 3-dB difficulty understanding speech in noise is more important than a 21-dB difficulty understanding speech in quiet conditions, and that tone thresholds and speech intelligibility in noise cannot be directly correlated^5^.

In the Brazilian clinical practice, the assessment of hearing capabilities in the presence of noise has been included in the protocol used to evaluate central auditory processing. An important factor that leads patients without peripheral auditory disorder to be referred to central auditory processing assessment is difficulty understanding speech in noisy or reverberative environments^7^. This test takes a long time (approximately three one-hour sessions), and is thus harder to be incorporated to a center's audiological testing routine.

Auditory processing disorder is not the only hearing alteration that may be missed in an audiogram. Alterations arising from the inner hair cells, the auditory nerve, and the efferent system (feedback alteration) may not result in disordered pure-tone sensitivity or speech recognition in quiet conditions, but can compromise speech recognition in noisy environments^8^.

Many studies have attempted to understand the conditions underlying central auditory processing disorders. Although hearing difficulty was the reason for patients to have their auditory processing assessed, many individuals submitted to these tests ended up having results within normal range and no further explanation for their complaints.

This study aimed to investigate self-reported hearing-related communication difficulties and compare pure-tone thresholds, speech recognition in quiet and noisy conditions, and brainstem auditory evoked potentials (BAEP) of adult subjects with normal audiograms and communication disorders and individuals with normal audiograms and no communication disorders.

## METHOD

Twenty patients aged between 16 and 49 years without specific middle-ear complaints and with A-sha-ped tympanogram tracings in both ears were enrolled in the study. This study was approved by the institution's Ethics Committee and given permit 046/2010. All patients signed an informed consent term.

Participants were divided into two groups: 1) case group - ten subjects (three males and seven females) with hearing thresholds of 25 dBNA and under in all tested frequencies who sought ENT care due to speech recognition impairment; 2) control group - ten subjects (three males and seven females) with hearing thresholds of 25 dBNA and under in all tested frequencies with no hearing complaints.

Case group subjects were referred to the study by ENTs and speech and hearing therapists. Sixteen subjects were referred within ten months. They were pre-selected based on their charts, and two were excluded for not meeting the enrollment criteria. The remaining 14 patients were contacted, but one refused to join the study while three were excluded due to altered audiometry results. Control subjects were paired for gender and age against case group individuals. At the time of enrollment they were asked about the presence of hearing complaints. Absence of complaints on the control group was confirmed through a specific protocol (APHAB - *Abbreviated Profile of Hearing Aid Benefit*). Subjects in the case group had their complaints confirmed through the same protocol.

In order to verify whether the pure-tone thresholds impacted the outcome of the tests, the data from study participants was grouped and analyzed based on a new criterion: one group with mean hearing thresholds at 250 and 500 Hz under 15 dB (G < 15 dB) and another group with mean hearing thresholds at 250 and 500 Hz of 15 dB and above (G ≥ 15 dB).

### Equipment and Procedures

#### Hearing impairment self-assessment scale

The tool used to quantify hearing complaints was the APHAB (Abbreviated Profile of Hearing Aid Benefit) self-assessment scale^9^. This scale was originally developed to quantify the subjective benefits provided by hearing aids, but it may be useful in quantifying auditory complaints connected to communication contexts. This tool is available in 16 languages and has been used in numerous studies. The Brazilian Portuguese version was translated by Almeida^10^.

Subject self-reports can be assessed based on four different situations represented in four APHAB sub-scales: EC (ease of communication), BN (background noise), RV (reverberation), and AV (aversiveness). The scores in the APHAB scale account for how often patients experience the issues related to each sub-scale as a percentage. Higher scores signify higher incidence of the issues related to each sub-scale. The global score of the APHAB scale is calculated as the mean value of the scores obtained in the EC, BN, and RV sub-scales. Sub-scale AV is not included in the APHAB global score as it is not related to communication situations. Thus, sub-scales EC, BN, and RV were considered to verify the presence of communication-related difficulties in case and control group subjects. The individual scores on each sub-scale were compared to APHAB scores of young adults subjectively deemed normal to characterize the complaints manifested by the subjects enrolled in the study.

#### Pure-tone audiometry

Hearing thresholds were analyzed for frequencies ranging between 250 and 8000 Hz, with the subjects inside an acoustic booth wearing TDH-39 earphones. A Madsen Electronics Midimate 622 audiometer calibrated as per standard ANSI 1969 was used. Speech recognition tests in quiet and noise were carried out with the same device.

#### Monaural sentence recognition test in quiet and noise

The tests were carried out with the subjects wearing TDH-39 earphones, and by playing the LSP^11^ - List of Sentences in Portuguese - with 10 sentences and noise within the range of speech recorded in independent channels. The recording was played in a Coby CD player coupled to the audiometer. Before the evaluation was initiated, the output level on each channel was calibrated using a VU-meter set at zero when a 1000 Hz tone was played. The presented signal (LSP) values were based on the speech values recorded and observed in the device's dial.

During the test, subjects were instructed to repeat whatever they could understand from the sentences played to them. They were not trained to cope with the sentences played in noise, and the groups' performances were compared in both situations: first in a test session in which they had not had previous contact with noise (right ear) and in a second session (left ear) after they had undergone the first test session with noise.

#### Sentence recognition ratio in quiet and noise

Subjects' right ears were initially presented the 10 sentences on list 1A at 65 dB without noise. Then list 1B was presented to the same ear at 65 dB with ipsilateral noise at 65 dB (S/N = 0 dB). Finally, list 2B was presented at 65 dB and ipsilateral noise at 70 dB (S/N = −5 dB). The same procedure was carried out for left ears, and the following sentence lists were presented in the same respective order: 3B, 4B, and 6B. A sentence recognition ratio was calculated based on the number of right answers, according to the instruments' instruction manual. Each correctly repeated sentence accounted for 10% of the ratio.

#### Brainstem auditory evoked potentials

Potentials were measured using a Contronic Masbe ATC Plus device. Auditory pathway integrity was assessed by the onset and reproducibility of waves I, III, V, and interpeak intervals I-III, I-V, III-V at 80 dBNA. Tests were carried out in an electrically and acoustically insulated room. Subjects had electrodes placed in their left and right ear mastoids. Click stimuli with altered polarity were presented at a rate of 17.1 clicks per second with an intensity of 80 dBNA to subjects wearing TDH-39 earphones.

#### Data treatment

As the samples were small and the data did not follow a normal distribution, the Mann-Whitney test was performed to verify the existence of in-group statistical differences. Correlation analysis was done using Pearson's r. Statistical analyses were done using the Statistical Package for the Social Sciences, version 17.0. Statistical significance was attributed when *p*< 0.05.

## RESULTS

### APHAB scale

All case group members had scores in at least one of the sub-scales related to communication situations (EC, BN, RV) above the 95^th^ percentile of the scale's normative values. In this group, eight (80%) individuals had scores above the 95^th^ percentile in sub-scale BN, seven (70%) in sub-scale RV, and six (60%) in sub-scale EC. Two participants (20%) had above normal scores in sub-scale AV (aversiveness). Control group participants had results below the 95h percentile in sub-scales EC, BN, and RV, but two (20%) individuals had scores above the 95^th^ percentile in sub-scale AV.

**Figure 1:**
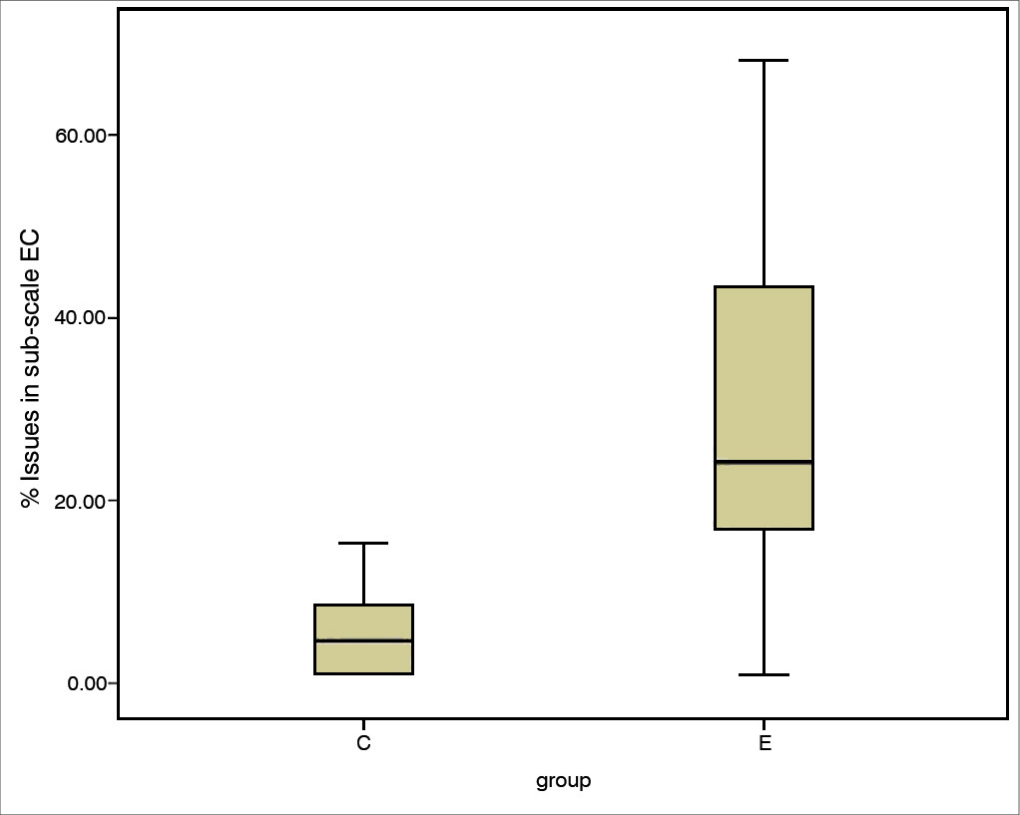
EC sub-scale scores (median, 1^st^ and 3^rd^ quartiles, and adjacent values) for case and control groups. EC: Ease of Communication; C: control group; E: case group.

**Figure 2:**
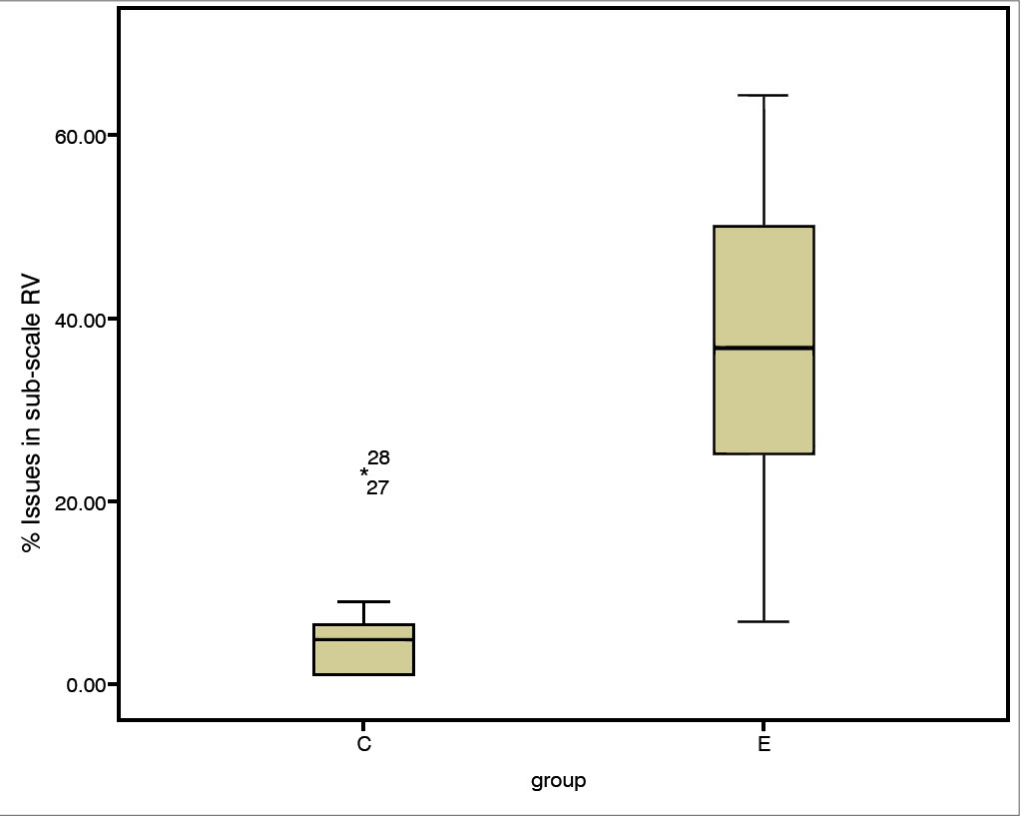
RV sub-scale scores (median, 1^st^ and 3^rd^ quartiles, and adjacent values) for case and control groups. C: control group; E: case group.

**Figure 3:**
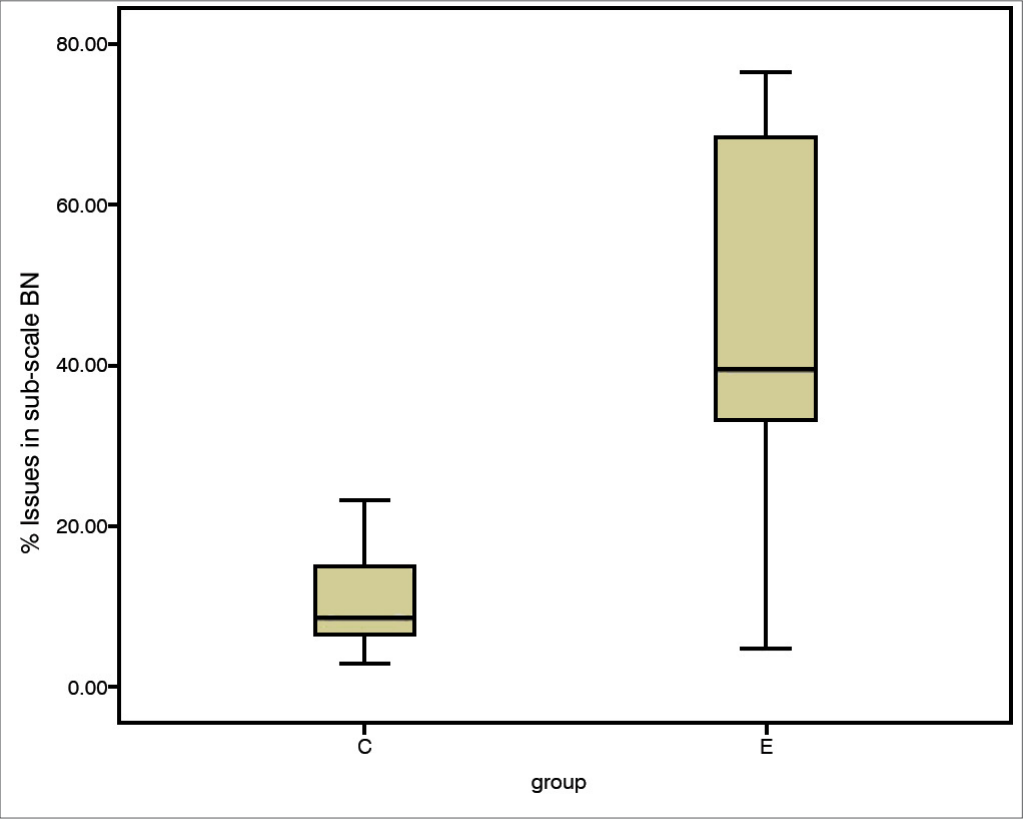
BN sub-scale scores (median, 1^st^ and 3^rd^ quartiles, and adjacent values) for case and control groups. C: control group; E: case group.

**Figure 4:**
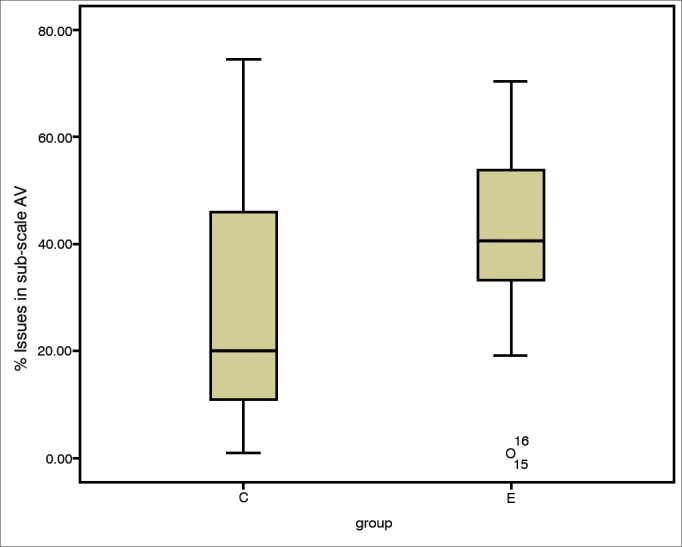
AV sub-scale scores (median, 1^st^ and 3^rd^ quartiles, and adjacent values) for case and control groups. C: control group; E: case group.

The incidence of communication difficulties varied between case and control subjects, as seen in scales EC (c^2^ = 12.45; *p* = 0.002), RV (c^2^ = 14.68; *p* = 0.001), BN (c^2^ = 10.37; *p* = 0.006), and AV (c^2^ = 2.46; *p* = 0.3). The Mann-Whitney test indicated that case group subjects had higher incidence rates of communication disorder in environments favorable to communication (Figure 1), EC (U = 10.50; *p* = 0.002), environments with reverberation (Figure 2), RV (U = 4.00; *p* = 0.000), and in noise (Figure 3), BN (U = 16.00; *p* = 0.009) than controls; no significant differences were seen in situations of aversive sound (Figure 4) between case and control group members (U = 33.5; *p* = 0.218).

### Percent recognition of sentences in quiet (PRSQ)

All participants had 100% PRSQ in both ears (*p* = 1.00).

### Percent recognition of sentences in noise (PRSN)

**Figure 5:**
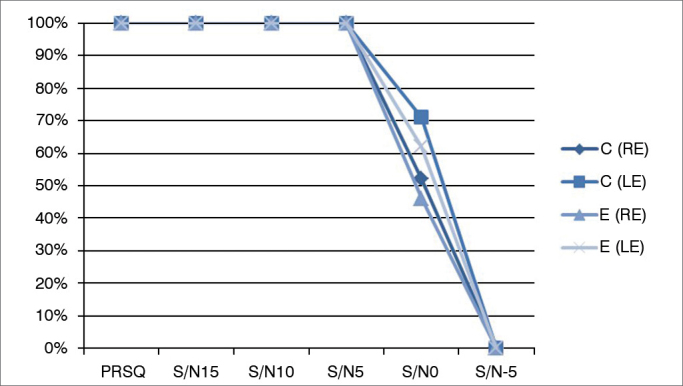
Comparison of median percent recognition of sentences in different signal to noise ratios in case and control group subjects. PRSQ: Percent Recognition of Sentences in Quiet; S/N: Signal to noise ratio in dB; C: control group; E: case group; RE: Right Ear; LE: Left Ear.

High levels of variability were observed in PRSN (N/S = 0 dB). The percentage of right answers in the control group ranged from 30% to 100%, and from 0% to 90% among case group subjects. The difference between groups was not statistically significant, either when right (c^2^ = 101.50; U = 46.50; *p* = 0.787) and left ears (c^2^ = 96.00; U = 41; *p* = 0.491), or both ears were compared (c^2^ = 388.50; U = 178.50; *p* = 0.557). At a signal to noise ratio of −5 dB, the percent recognition rates among controls ranged from 0% to 10%, while case group individuals presented a rate of 0%. The Mann-Whitney test revealed no differences between groups in PRSN at a S/N ratio of −5 dB (*p* = 0.435) (Figure 5).

### Pure-tone thresholds

**Figure 6:**
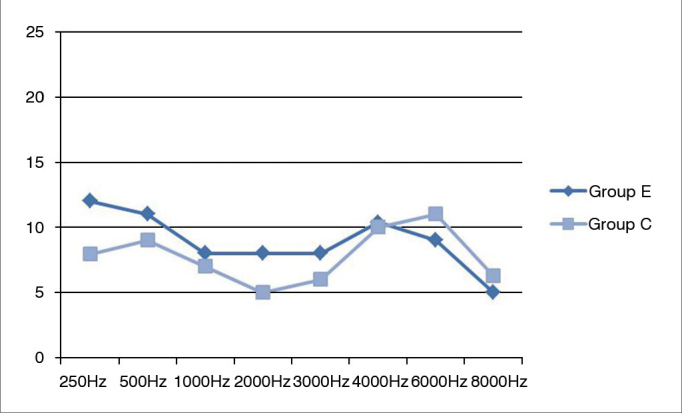
Mean pure-tone thresholds at 250 to 8000 Hz in case and control groups. C: control group; E: case group.

The differences in the pure-tone thresholds of case and control group individuals were not statistically significant (Figure 6).

### Brainstem auditory evoked potentials

Latencies in the onset of waves I (generated in the distal portion of the cochlear nerve), III (generated in the cochlear nuclei), and V (generated by the lateral lemniscus) from the introduction of acoustic stimuli, and interpeak intervals I-III, I-V, and III-V of case and control group members were within normal range. The Wilco-xon signed-rank test showed no statistically significant differences between the latency and interpeak interval values of case and control group subjects. The lowest observed *p-*value was 0.609.

Given the lack of difference between ears, the Mann-Whitney test was used to check whether in-group differences were statistically significant, only to find they were not (lowest *p*-value of 0.417). The Kolgomorov-Smirnov test confirmed the absence of differences between case and control groups (lowest *p-*value of 0.329).

Pearson's r correlation analysis:

APHAB *vs.* PRSQ. No significant correlations were observed between hearing complaints and performance in speech recognition tests in quiet and noise.

BAEP *vs.* APHAB. An inverse correlation was observed between AV scores and interpeak interval I-V (r = −0.319; *p* = 0.045), i.e., the lower the I-V interpeak interval the higher the AV score.

Pure-tone thresholds *vs.* APHAB. Greater thresholds were correlated to higher scores in sub-scales related to communication situations. The thresholds at 250 and 500 Hz were within normal range (≤ 25 dB), but were correlated to sub-scales ease of communication, reverberation, background noise, and global APHAB scores. Thresholds at 1000 Hz were correlated to ease of communication, and thresholds at 2000 Hz were correlated to ease of communication and reverberation. All correlations were positive, i.e., higher thresholds at 250, 500, and 1000 Hz were correlated to higher incidence rates of communication difficulties (Table 1). AV-related complaints were inversely correlated to control group subject scores. Lower thresholds at 500 Hz meant higher AV scores (r = −0.449; *p* = 0.047).

**Table 1:** Correlation between pure-tone thresholds in each tested frequency and APHAB scale scores for case and control groups.

APHAB	Thresholds per frequency (Hz)								
		250	500	1000	2000	3000	4000	6000	8000
EC	r	0.621*	0.528*	0.314*	0.378*	127	–0.089	–0.128	0.088
	*p*	0.000	0.000	0.049	0.016	0.435	0.587	0.432	0.590
RV	*r*	0.459*	0.389*	0.203	0.352*	0.239	0.089	–0.008	–0.013
	*p*	0.003	0.013	0.208	0.025	0.138	0.585	0.960	0.935
BN	*r*	0.349*	0.498*	0.155	0.086	0.098	0.043	0.047	–0.025
	*p*	0.028	0.001	0.338	0.596	0.549	0.792	0.772	0.876
AV	r	–0.032	0.075	–0.044	–0.222	–0.278	–0.011	0.068	–0.048
	*p*	0.845	0.644	0.786	0.168	0.082	0.944	0.678	0.767

EC: Ease of Communication; BN: Background Noise; RV: Reverberation; AV: Aversiveness; APHAB: Abbreviated Profile of Hearing Aid Benefit.

The analyses of test results based on pure-tone thresholds showed, according to the Mann-Whitney test, that individuals with thresholds of 15 dB and above reported statistically more communication difficulties than subjects with thresholds under 15 dB (Table 2). The groups were not statistically different in terms of recognition of sentences in noise, brainstem evoked auditory potentials, and acoustic reflexes.

**Table 2:** Difference between groups G < 15 dB e G ≥ 15 dB in APHAB scores.

Medida	EC	RV	BN	AV	APHAB
U	37.000**	59.000**	68.000*	127.000	50.000**
*p*	0.000	0.004	0.010	0.472	0.002

EC: Ease of communication; BN: Background Noise; RV: Reverberation; AV: Aversiveness; APHAB: Abbreviated Profile of Hearing Aid Benefit.

## DISCUSSION

The APHAB scale has been successfully used as an instrument to verify the subjective benefit offered by hearing rehabilitation, including hearing aids, cochlear implants, and auditory training^12^. However, no other studies are known to have used this instrument to verify and characterize self-reports of adult subjects with hearing complaints and without audiogram-verified alterations, which limited the comparison of the data shown in this study to the data published by other authors. Even the study performed with the purpose of establishing normative values for young adults, the enrollment criteria was subjects being “subjectively normal”^9^.

Comparisons against the scale's normative values indicated that all case group individuals had scores above the 95^th^ percentile in at least one of the sub-scales related to contexts of communication. Case and control group subjects were statistically different in relation to their auditory complaints. Despite the confirmation of the difference in hearing complaints, the results of the tests run in noise were not statistically different between the groups.

The enormous variability observed in the performance of case and control group subjects in recognition of sentences in noise may explain the absence of statistical differences between the groups. The variability seen in the case group was due to sample heterogeneity, a factor that could have been greatly reduced by more stringent enrollment criteria to eliminate the interference of other variables that affect speech recognition in noise, such as memory and attention disorders.

Statistical analysis pointed to the existence of significant differences between ears in the sentence recognition tests in noise of case and control group individuals, with left ears performing better than right ears. This difference may be related to the fact that right ears were tested first for sentence recognition in noise, and left ears may have benefitted from a learning effect. Other authors have also seen improved performance in the second ear tested, regardless of side^13^^,^^14^.

This study did not aim to verify the differences in performance between right and left ears. If that were the case, the study would have to be designed in a way to eliminate or mitigate the interferences of the learning effect by, for example, including bilateral ear training before running the tests. Dichotic tests carried out in studies that looked into differences between left and right ears have suggested that right ears perform better^15^^,^^16^. Such difference reflects the functional differences between brain hemispheres and the fact that right ears send more impulses to the left side of the brain, which is specialized in language processing^17^.

The significant variability in the performance of individuals from both groups may explain the absence of statistical differences between them. Therefore, correlation analysis was carried out on the combined results of case and control group subjects in the sentence recognition test in noise and their scores in the APHAB scale, in order to verify whether participants who performed better in speech recognition in noise also has lower APHAB scores. However, the analysis confirmed that self-reports were not correlated to performance in speech recognition tests in noise. Not even the BN sub-scale scores were correlated to speech recognition tests in noise when both groups were analyzed.

An explanation for the absence of a correlation is the lack of awareness from control group members of the possible hearing impairments they may have. Several studies have analyzed the accuracy of hearing loss complaints and absence of complaints and found that approximately 20% of the individuals with hearing claim not to have hearing impairment^18^, ^19^, ^20^. It is possible that a larger sample could have minimized the effects of lack of awareness of hearing loss, to the elicit differences between the groups on the studied variables.

Authors assessed the efficiency of central auditory function in elderly subjects who reported they could hear well and found that - despite the self-reports of good hearing − 60% of the studied individuals had hearing loss and significant incidences of impaired central auditory function^20^. In our study, the performance variability in the speech recognition test in noise seen in both groups, connected to the fact that some controls performed significantly worse than case group individuals, reinforce the idea that the lack of awareness of one's hearing impairment may have contributed to the absence of a correlation between self-reports and speech recognition in noise.

Both false negatives and false positives are possible in patient complaints. Psychosocial matters may be at play in the genesis of the complaint, as in chronic complaint disorders, in which patients go to various physicians complaining of multiple symptoms -none of which supported by examination and testing^21^. However, the investigation of the condition underlying the complaint is required and the findings in this study ratify this position, specifically when it comes to the variability seen in the performance of subjects in the speech recognition tests in noise.

This study did not aim to see whether subjects had central auditory processing disorders. Once the standard for normal results in audiological examination is based in criteria that may not be sensitive to capture minor alterations, we tried to verify if, even in the presence of normal test results, the groups with statistical differences in their complaints would perform differently in speech recognition tests in noise - and they did not. It is worth pointing out that one cannot state, based on the tests carried out in this study alone, that individuals reporting hearing impairment do not really experience auditory difficulties in their lives. One may only conclude that the reported difficulty was not confirmed in a structured setting. Tests were monaural (signal and noise played into one ear at a time), in a situation that differs greatly from the everyday life of the subjects enrolled in the study, in which dichotic hearing is used more frequently. Given the variety of environments and noises associated with different types of discourse, one cannot assume that the ability to understand speech in all circumstances can be measured by one single speech recognition test in noise^2^.

The same logic may be applied to the interpretation of the absence of a correlation between hearing complaints related to communication contexts and the sentence recognition rate in quiet conditions. Can self-report reflect the actual difficulty subjects experience? Can one single test measure individual performance? Authors indicate that questionnaires usually fail to describe a given situation with the same level of control and precision obtained in lab experiments^1^. By the same token, these authors draw our attention to the fact that lab experiments are not better at accurately portraying the actual magnitude of one's hearing difficulty. Lindley stressed that self-assessment instruments contribute to the verification of hearing performance, once it would be impossible to simulate all situations of daily life in a laboratory^22^.

Many authors have used absence of auditory complaints as an enrollment criteria, but there are no published validated instruments to analyze this criterion. Analysis is usually done by questions such as ‘Do you have any hearing complaints?' The findings in this study suggest the APHAB scale is an appropriate instrument to capture the presence and absence of hearing complaints, principally as it allows for comparisons against normative values based in subjectively normal hearing adults.

Considering the comparison of hearing thresholds, no differences were found between case and control group subjects. Even with the enrollment criterion of thresholds of 25 dBNA and under, statistical differences could have been found between the groups.

Sentence recognition tests in noise failed to detect the underlying conditions related to the reported hearing difficulties in communication situations. However, a positive correlation was found between pure-tone thresholds and scores in the scales connected to self-reported difficulties, i.e., higher thresholds meant higher scores in everyday life hearing difficulties, although they were within normal range.

This finding shows how broad the range of normality can be, as also indicated by other authors in a study that compared thresholds between 250 and 1600 Hz of young adults and elderly subjects with hearing thresholds of 25 dB and below in frequencies between 250 and 8000 Hz^23^. This study revealed significantly higher pure-tone thresholds in the older group, although all subjects had normal audiograms. In the above mentioned study complaints were not significantly correlated with thresholds, but the authors suggested that a more sensitive instrument may portray more accurately the aspects connected to subject complaints and, thus, reveal the impact of this variable.

In this study, although the thresholds of case and control groups at 250 and 500 Hz were within normal range, there was a positive correlation between thresholds and sub-scales EC, RV, BN, and the global APHAB score. Control group subject thresholds were inversely and significantly correlated to the AV sub-scale. Thus, self-reported hearing difficulty in communication contexts was associated with higher thresholds, while greater difficulty hearing aversive sounds was correlated with lower thresholds (greater sensitivity).

The data from both groups were rearranged based on low-frequency tone sensitivity. One of the groups featured individuals with mean thresholds at 250 and 500 Hz under 15 dB (G < 15 dB) while the other included subjects with mean thresholds of 15 dB and above (G ≥ 15 dB). The group with less low-frequency sensitivity reported difficulties in communication situations more frequently than the group with thresholds under 15 dB.

The indication that higher pure-tone thresholds at lower frequencies correlate to higher scores of self-reported difficulty calls for reflections on the standard of normality. Despite the excessive flexibility of this criteria, the fact that outer hair cell alterations lessen auditory sensitivity more frequently at higher frequencies makes one wonder why a positive correlation between thresholds and self-report of hearing difficulty was not observed at higher frequencies. People with altered sensitivity at higher frequencies combined with normal sensitivity at lower frequencies usually report normal sensitivity along with difficulty understanding speech^24^.

Thresholds at 1000 Hz were also correlated with sub-scale EC, while thresholds at 2000 Hz were correlated to sub-scales EC and RV. Higher thresholds at 250, 500, 1000 and 2000 Hz meant higher incidence of communication difficulties, thus reinforcing the predictive value of the APHAB scale.

In Brazil, the classification proposed by Lloyd & Kaplan is the most widely used scheme to assess the degree of hearing loss in adult subjects. Normal hearing in this classification is assigned to subjects whose mean air conduction thresholds at 500, 1000 and 2000 Hz is of 25 dBNA and under^25^. The *Bureau Internacional d'Audio Phonologie*(BIAP), an institution that congregates several European associations with the main purpose of guiding health care workers in the region, considers that individuals with normal hearing have mean thresholds of 20 dBNS and under at frequencies between 500 and 4000 Hz^25^. Individuals with mean thresholds of 21 dBNA and under are deemed to have hearing loss. The findings of this study support the indication of more stringent criteria for normal hearing, as recommended by the BIAP.

Authors using the APHAB scale reported inconsistencies in the data related to sub-scale AV^26^. A study compared the American and the Polish version of the APHAB scale, and found that only the AV sub-scale in the Polish scale produced different scores from the original US version^27^.

Normative values in the AV scale present the higher levels of variation in subject responses, with the 5^th^ percentile being equal to 2 and the 95^th^ percentile equal to 54 - the highest value in the 95^th^ percentile of the normal distribution. The 95^th^ percentiles of the other sub-scales are: 21 (EC), 29 (RV), and 34 (BN).

In this study, sub-scale AV was the only not to present differences between case and control group subjects. However, when analyzing the correlation between this sub-scale and the other tests, we found that lower I-V interpeak intervals meant higher scores in the AV sub-scale. The analysis of interpeak intervals allows the identification of possible alterations in the trajectory of the acoustic stimulus along the auditory pathway. As wave I is generated in the distal portion of the auditory nerve and wave V starts at the inferior colliculus, increased I-V interpeak intervals suggest differences in case and control group auditory processing. This finding is worthy of further investigation.

Indeed, in addition to the variables analyzed in this study, others have been indicated by a number of authors. Caporali & Silva, for instance, observed that speech recognition in noise is a task that requires the use of memory and selective attention, as individuals need to focus their attention to the sound of interest and evoke information while they ignore irrelevant information (noise)^13^. The physiology of the use of memory and selective attention may be analyzed through medium and long latency evoked potentials occurred after BAEP^28^. This test is considered to be one of the best to assess the nervous system and auditory processing disorders, as it presents important information on auditory processing alterations, cognitive and language disorders.

Future research could, therefore, look into the auditory pathways after the brainstem and compare the results of case and control groups in terms of medium and long latency evoked potentials to present evidences on the neural substrate connected to communication difficulties.

## CONCLUSION

Case group subjects reported statistically more communication difficulties than their control group counterparts. However, when group test results were compared, no statistically significant differences were found in the latencies of waves generated in the distal portion of the cochlear nerve, in the cochlear nuclei, and in the lateral lemniscus.

There certainly are many variables involved in speech recognition, specifically in environments with reduced extrinsic redundancy. The differences in pure-tone thresholds within normal range affect the self-report of communication difficulties. However, the differences in complaint self-reporting were not reproduced statistically in pure-tone thresholds. Thus, it was not possible to correlate the variables connected to auditory function from the cochlear nerve to the brainstem with differences in complaint self-reporting of case and control group subjects.
